# COPS7B Drives Malignant Progression of Glioblastoma Through Translational Upregulation of the Downstream Functional Effector CLU

**DOI:** 10.3390/ijms27156976

**Published:** 2026-08-03

**Authors:** Jiarui Li, Meiling Zhang, Fan Xu, Xi Liu, Yunting Le, Zhaozhan Fan, Chen Chen, Liyuan Guo, Shuoshuo Wang

**Affiliations:** 1 Department of Pathology, School of Basic Medical Science, Harbin Medical University, Harbin 150081, China; lijiarui@hrbmu.edu.cn (J.L.);; 2 Ordos Clinical Medical College, Inner Mongolia Medical University, Ordos 017000, China

**Keywords:** glioblastoma, COPS7B, CLU, translation, post-transcriptional regulation

## Abstract

Glioblastoma (GBM) is a highly aggressive primary malignant brain tumor, featuring diffuse infiltrative growth and poor clinical outcomes, which underscores the need to delineate the molecular mechanisms driving its malignant progression. COP9 signalosome subunit 7B (COPS7B), a core component of the conserved COP9 signalosome complex, is significantly upregulated in GBM tissues; however, its biological function and regulatory mechanism in GBM remain largely elusive. Here, we found that elevated COPS7B expression was positively correlated with glioma pathological grade and adverse prognosis in histologically and molecularly confirmed GBM patients. Functional assays demonstrated that COPS7B markedly promoted the proliferation, migration, and invasion of GBM cells in vitro, while COPS7B knockdown exerted the opposite suppressive effects. Mechanistically, we identified clusterin (CLU) as a key downstream functional effector of COPS7B in GBM. COPS7B upregulated CLU protein abundance by enhancing the translation efficiency of CLU mRNA, without altering its transcriptional level or protein stability. Functional rescue experiments further confirmed that CLU is indispensable for COPS7B-mediated malignancy-driving phenotypes in GBM, and transcriptomic analysis revealed that the progression-promoting effect of CLU was tightly associated with the activation of tumor-related signaling cascades, including the ERK and MAPK pathways, as well as the regulation of cell growth, invasion, and migration. Collectively, this study not only reveals a critical role of COPS7B in driving GBM malignant progression but also delineates a novel COPS7B-CLU regulatory axis that drives GBM aggressive phenotypes via activation of mitogenic signaling, suggesting candidate targets for further translational investigation.

## 1. Introduction

Glioblastoma (GBM) is among the most lethal primary malignant tumors of the adult central nervous system. Population-based registry data report a median overall survival (OS) of approximately 15 months, with 1-year, 3-year, and 5-year overall survival rates of ~41%, ~11%, and merely 7.2%, respectively [1]. Despite decades of therapeutic optimization, clinical outcomes for GBM patients remain stagnant, largely due to the incomplete understanding of the molecular mechanisms driving its malignant progression [2,3].

The COP9 signalosome (CSN) is a highly conserved multiprotein complex that canonically regulates cullin-RING E3 ubiquitin ligase (CRL) activity to control ubiquitin–proteasome system (UPS)-mediated protein degradation [4]. The mammalian CSN holoenzyme consists of eight core subunits (CSN1–CSN8), among which COP9 signalosome subunit 7B (COPS7B) is essential for CSN complex assembly and the maintenance of full holoenzyme function [5]. Accumulating evidence has implicated dysregulated CSN subunits in tumor progression across multiple human malignancies; however, the vast majority of prior studies have focused on their canonical deneddylation-dependent function in modulating proteasomal protein degradation [6,7]. Notably, previous studies have shown that COPS7B has a clear biological function that is independent of CSN. Besides its structural function within the CSN complex, COPS7B can localize to ribosomes and directly regulate ribosome biogenesis and global mRNA translation in a manner independent of the CSN holoenzyme and the ubiquitin–proteasome degradation pathway [8]. Dysregulated COPS7B expression drives malignant progression in multiple malignancies, including colorectal carcinoma, hepatocellular carcinoma, and renal cell carcinoma, suggesting it may act as a broad oncogenic regulator [8,9,10]. Given its central role in controlling oncogenic protein expression at post-transcriptional levels, targeted modulation of COPS7B holds potential as an interventional strategy for relevant malignancies. However, to date, no studies have investigated the expression, clinical significance, or biological function of COPS7B in GBM, and whether its non-canonical translational regulatory activity contributes to GBM malignant progression remains completely unexplored.

In this study, we aimed to systematically elucidate the clinical significance, biological function, and underlying molecular mechanism of COPS7B in GBM, with a particular focus on its non-canonical role in translational regulation. Uncovering the COPS7B-mediated regulatory network in GBM may provide novel prognostic biomarkers and candidate interventional targets for this devastating malignancy.

## 2. Result

### 2.1. COPS7B Is Upregulated in Glioma Tissues and Correlates with Higher Tumor Grade and Adverse Prognosis in GBM Patients

**Figure 1:**
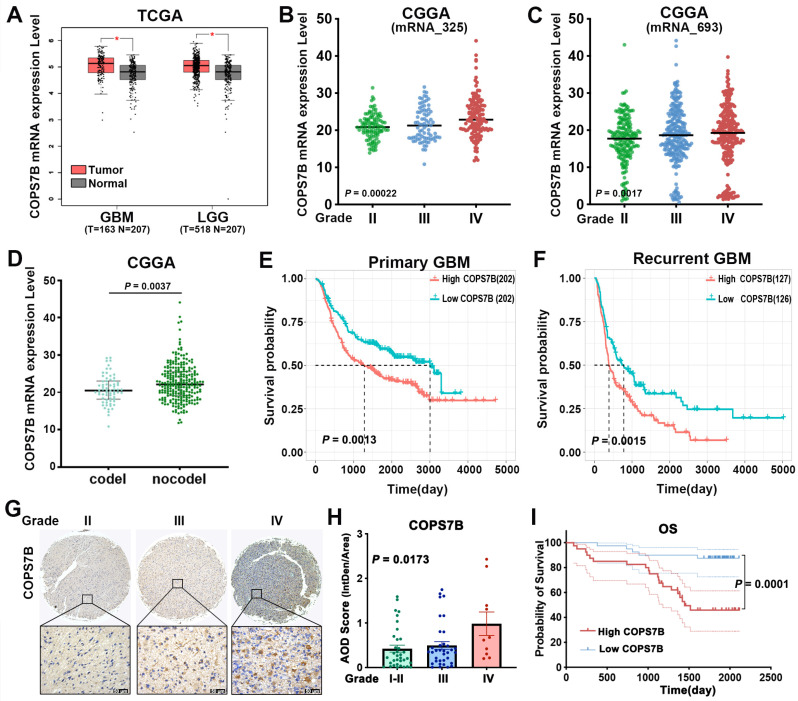
COPS7B expression is elevated in glioma and predicts poor survival in GBM. (**A**) COPS7B mRNA expression in normal brain tissues (GTEx) and glioma tissues (TCGA-LGG and TCGA-GBM). Statistical significance was determined by one-way ANOVA followed by Tukey’s post hoc test. *, *p* < 0.05. (**B**) Scatter plot showing COPS7B mRNA expression across WHO glioma grades (II, III, IV) in the CGGA 325 cohort. Statistical significance was determined by one-way ANOVA followed by Tukey’s post hoc test. (**C**) Scatter plot showing COPS7B mRNA expression across glioma grades (II, III, IV) in the CGGA 693 cohort. Statistical significance was determined by one-way ANOVA followed by Tukey’s post hoc test. (**D**) Scatter plot showing COPS7B mRNA expression in 1p/19q codeleted and non-codeleted glioma samples from the CGGA cohort. Statistical significance was determined by two-tailed Student’s *t*-test. (**E**) Kaplan–Meier survival analysis showed the correlation between COPS7B mRNA expression level and OS in patients with primary GBM cancer from the CGGA cohort. Statistical significance was determined by the log-rank test. (**F**) Kaplan–Meier survival analysis showed the correlation between COPS7B mRNA expression level and OS rate of patients with recurrent GBM cancer from the CGGA cohort. Statistical significance was determined by the log-rank test. (**G**) Representative IHC staining images of COPS7B in glioma microarray. Magnification, ×400. Scale bar, 50 μm. (**H**) Quantitative analysis of IHC microarray staining intensity (grade I–II, *n* = 35; III, *n* = 35; IV, *n* = 10). Data are presented as mean ± SD. Statistical significance was determined by one-way ANOVA followed by Tukey’s post hoc test. (**I**) Kaplan–Meier OS analysis showing the association between COPS7B protein expression and OS in glioma patients from the IHC tissue microarray (Blue lines: low expression group, *n* = 40; red lines: high expression group, *n* = 40). Statistical significance was determined by the log-rank test.

We first examined the mRNA expression of COPS7B across GBM, lower-grade glioma (LGG), and normal brain tissues using transcriptomic data from the GEPIA web server. The results showed that COPS7B mRNA levels were significantly higher in glioma tissues than in normal brain controls (Figure 1A). To investigate the association between COPS7B expression and glioma malignant progression, we further analyzed mRNA expression data with matched clinical annotations from the CGGA dataset and found that COPS7B expression was positively correlated with increasing WHO pathological grade of glioma (Figure 1B,C). In addition, COPS7B expression was significantly elevated in 1p/19q non-codeleted gliomas compared with 1p/19q codeleted tumors, a molecular subtype linked to more aggressive clinical behavior (Figure 1D). Kaplan–Meier survival analysis using the same cohort revealed that both primary and recurrent GBM patients with high COPS7B mRNA expression had significantly shorter OS than those with low expression (Figure 1E,F).

To validate these transcriptomic findings at the protein level in clinical specimens, we performed immunohistochemical staining (IHC) on a glioma tissue microarray. In line with the mRNA expression pattern observed in the CGGA cohort, COPS7B protein expression was positively associated with WHO pathological grade, with progressively higher staining intensity detected in higher-grade gliomas (Figure 1G,H). Furthermore, survival analysis based on IHC scoring demonstrated that GBM patients with high COPS7B protein abundance exhibited significantly poorer OS than patients with low expression (Figure 1I). Collectively, these data from both public transcriptomic datasets and an independent clinical tissue cohort indicate that COPS7B is upregulated in GBM, and its elevated expression is associated with aggressive clinicopathological features and unfavorable patient outcomes.

### 2.2. COPS7B Promotes Proliferation, Migration, and Invasion of GBM Cells In Vitro

**Figure 2:**
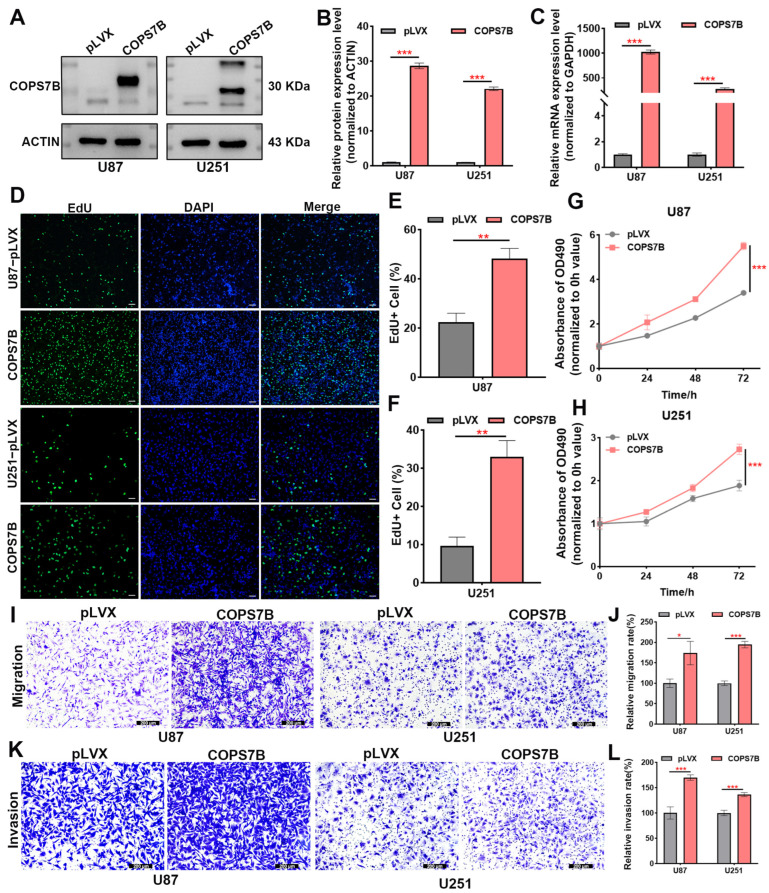
COPS7B overexpression potentiates the malignant progression. (**A**) Representative Western blot images showing COPS7B protein levels in control and COPS7B-overexpressing U251 and U87 cells. (**B**) Quantitative analysis of COPS7B protein levels in U251 and U87 cells with COPS7B overexpression. Data are presented as mean ± SD from three independent biological replicates. Statistical significance was determined by two-tailed Student’s *t*-test. ***, *p* < 0.001. (**C**) Relative COPS7B mRNA levels in U251 and U87 cells detected by RT-qPCR. Data are presented as mean ± SD from three independent biological replicates. Statistical significance was determined by two-tailed Student’s *t*-test. ***, *p* < 0.001. (**D**–**F**) The effects of COPS7B overexpression on cell proliferation in U87 and U251 cells were evaluated by EdU assays. Data are presented as mean ± SD from three independent biological replicates. Statistical significance was determined by two-tailed Student’s *t*-test. **, *p* < 0.01. (**G**,**H**) The effects of COPS7B overexpression on cell proliferation in U87 and U251 cells were evaluated by MTT assays. All cells were subjected to MTT analysis at the indicated time points. Data are presented as mean ± SD from 3 independent biological replicates. Statistical significance was determined by two-way ANOVA followed by Tukey’s post hoc test. ***, *p* < 0.001. (**I**–**L**) The effect of COPS7B overexpression on the migration of U251 and U87 cells using the migration and invasion assay. Data are presented as mean ± SD from three independent biological replicates. Statistical significance was determined by two-tailed Student’s *t*-test. *, *p* < 0.05; ***, *p* < 0.001. Magnification, ×100. Scale bar, 200 μm.

To investigate the biological function of COPS7B in GBM, we established stable COPS7B-overexpressing and COPS7B-knockdown cell lines in U251, U87, and LN229 GBM cells via lentiviral transduction. The overexpression and knockdown efficiencies were verified by Western blot and RT-qPCR (Figure 2A–C, Figures S1A–C and S2A). MTT and EdU incorporation assays showed that COPS7B overexpression significantly enhanced the proliferative capacity of the GBM cell lines, whereas COPS7B knockdown significantly suppressed cell proliferation (Figure 2D–H, Figures S1D–G and S2B–D). We then examined the effect of COPS7B on cell migratory and invasive abilities using Transwell assays. Consistent with the proliferation results, COPS7B overexpression significantly increased the number of migrated and invaded cells, while COPS7B knockdown significantly attenuated these malignant phenotypes (Figure 2I–L, Figures S1H–K and S2E–H). Taken together, these in vitro functional data demonstrate that COPS7B drives the proliferative, migratory, and invasive phenotypes of GBM cells.

### 2.3. Identification of CLU as a Key Downstream Effector of COPS7B via Quantitative Proteomic Screening

**Figure 3:**
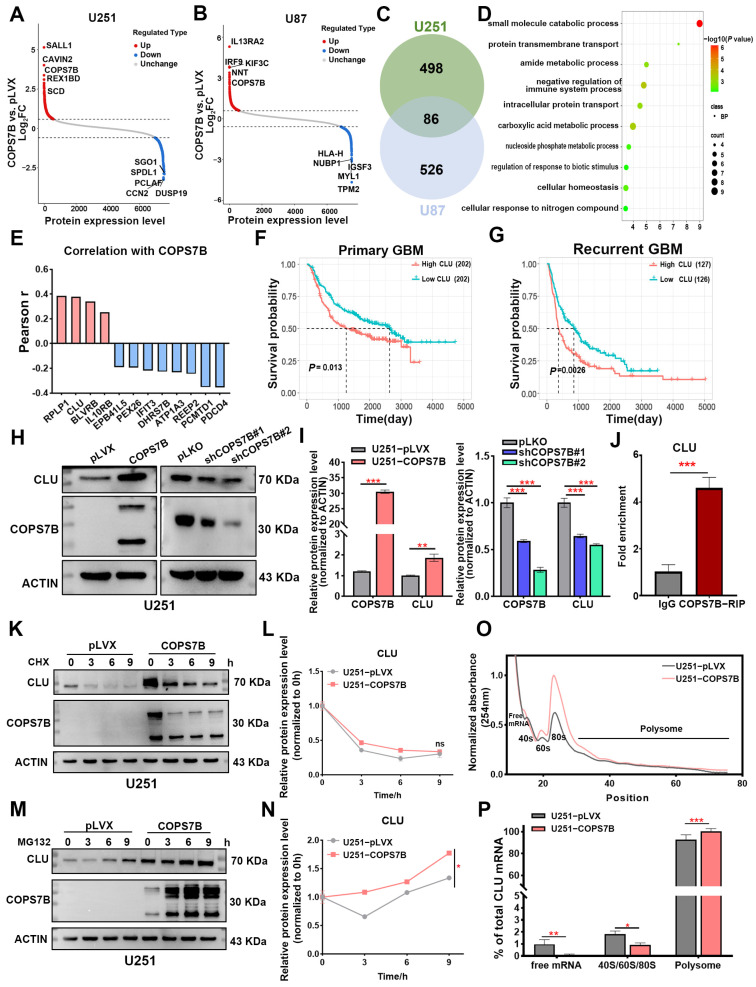
Screening the key downstream target genes of COPS7B that promote GBM. (**A**,**B**) Differentially expressed proteins in U251 and U87 cells after COPS7B overexpression were identified by LC-MS/MS. Red: upregulated proteins; blue: downregulated proteins; gray: proteins with no significant changes. (**C**) The intersection of differentially expressed proteins in U87 and U251 cells after COPS7B overexpression. (**D**) GO enrichment analysis was performed on the proteins up-regulated in COPS7B overexpression group. (**E**) Correlation analysis with COPS7B and target genes in the CPTAC database. (**F**) Kaplan–Meier survival analysis showed the correlation of the expression level of CLU and OS rate of patients with primary GBM cancer. Statistical significance was determined by the log-rank test. (**G**) Kaplan–Meier survival analysis showed the correlation between the expression level of CLU and OS rate of patients with recurrent GBM cancer. Statistical significance was determined by the log-rank test. (**H**) Representative Western blot images showing CLU and COPS7B protein levels in COPS7B-overexpressing and COPS7B-knockdown U251 cells and corresponding control cells. (**I**) Quantifications of Western blotting of the expression of CLU and COPS7B in U251 cells. Data are presented as mean ± SD from 3 independent biological replicates. Statistical significance was determined by one-way ANOVA followed by Tukey’s post hoc test. **, *p* < 0.01; ***, *p* < 0.001. (**J**) RIP-qPCR assay validating the direct interaction between COPS7B protein and CLU mRNA. Data are presented as mean ± SD from 3 independent biological replicates. Statistical significance was determined by two-tailed Student’s *t*-test. ***, *p* < 0.001. (**K**,**L**) The protein degradation levels of CLU in the overexpressing COPS7B group and the control group were statistically analyzed, and the CLU expression levels at 0 h were standardized. Data are presented as mean ± SD from three independent biological replicates. Statistical significance was determined by two-way ANOVA. ns, means no significant. (**M**,**N**) The protein synthesis levels of CLU in the overexpressing COPS7B group and the control group were statistically analyzed, and the CLU expression levels at 0 h were standardized. Data are presented as mean ± SD from 3 independent biological replicates. Statistical significance was determined by two-tailed Student’s *t*-test. *, *p* < 0.05. (**O**,**P**) Polysome profiling assay was performed in U251 COPS7B overexpression and control cells. The lysates of the COPS7B overexpression and control groups were separated into fractions using sucrose density gradients. The distribution of CLU mRNA was determined using RT-qPCR in the free mRNA, 40S/60S, 80S, and polysome fractions. Results were calculated as a percentage of the total RNA. Data are presented as mean ± SD from 3 independent biological replicates. Statistical significance was determined by two-tailed Student’s *t*-test for each fraction. *, *p* < 0.05; **, *p* < 0.01; ***, *p* < 0.001.

To delineate the molecular mechanism underlying the malignancy-driving function of COPS7B in GBM, we performed data-independent acquisition (DIA)-based quantitative proteomic profiling of COPS7B-overexpressing GBM cells and paired control cells to screen for differentially regulated proteins (Supplementary Table S2). In U251 cells, 584 proteins were significantly upregulated upon COPS7B overexpression; in U87 cells, 612 proteins were significantly upregulated (Figure 3A,B). Venn intersection analysis identified 86 proteins that were consistently and significantly upregulated in both cell lines (Figure 3C). GO functional enrichment analysis showed that these overlapping upregulated proteins were mainly enriched in biological processes including protein trafficking and cellular homeostasis (Figure 3D).

To further prioritize clinically relevant downstream effectors, we analyzed the correlation between COPS7B protein levels and the abundance of these 86 candidate proteins using the CPTAC GBM proteomic dataset. Four proteins—RPLP1, CLU, BLVRB, and IL10RB—showed significant positive correlation with COPS7B protein expression in clinical GBM tissues (Figure 3E; Supplementary Table S3). Among these candidates, CLU drew our attention because its high expression was closely associated with poor prognosis in both primary and recurrent GBM patients in the CGGA cohort, suggesting its potential as a functional mediator of COPS7B-driven malignancy (Figure 3F,G and Figure S3A–C).

We next validated the regulatory effect of COPS7B on CLU expression via Western blot and RT-qPCR. In both U251 and U87 cell lines, COPS7B overexpression significantly increased CLU protein levels, whereas COPS7B knockdown markedly reduced CLU protein abundance (Figure 3H,I and Figure S3F,G). However, RT-qPCR analysis showed no significant alteration in CLU mRNA levels upon COPS7B overexpression or knockdown (Figure S3D,E), indicating that COPS7B regulates CLU expression at the post-transcriptional level. RNA immunoprecipitation coupled with qPCR (RIP-qPCR) further demonstrated that COPS7B could bind to CLU mRNA, providing direct molecular evidence supporting this post-transcriptional regulatory mode (Figure 3J).

Given the canonical function of the COP9 signalosome in protein degradation through the ubiquitin–proteasome pathway, we subsequently examined whether COPS7B regulates CLU protein stability. When de novo protein synthesis was blocked by the translation inhibitor cycloheximide (CHX), the degradation rate of CLU protein showed no significant difference between COPS7B-overexpressing and control cells, ruling out regulation at the protein stability level. In contrast, treatment with the proteasome inhibitor MG132 to block endogenous protein degradation led to significantly faster accumulation of CLU protein in COPS7B-overexpressing cells, indicating that COPS7B promotes the de novo synthesis of CLU rather than affecting its protein stability (Figure 3K–N). Furthermore, polysome profiling showed that COPS7B overexpression enhanced global mRNA translation efficiency, which supporting the mechanism that COPS7B upregulates CLU protein abundance by boosting the translation efficiency of CLU mRNA (Figure 3O,P). Collectively, these data demonstrate that CLU is a downstream target of COPS7B in GBM cells, and that COPS7B upregulates CLU expression primarily at the translational level.

### 2.4. CLU Knockdown Suppresses Proliferation, Migration, and Invasion of GBM Cells

**Figure 4:**
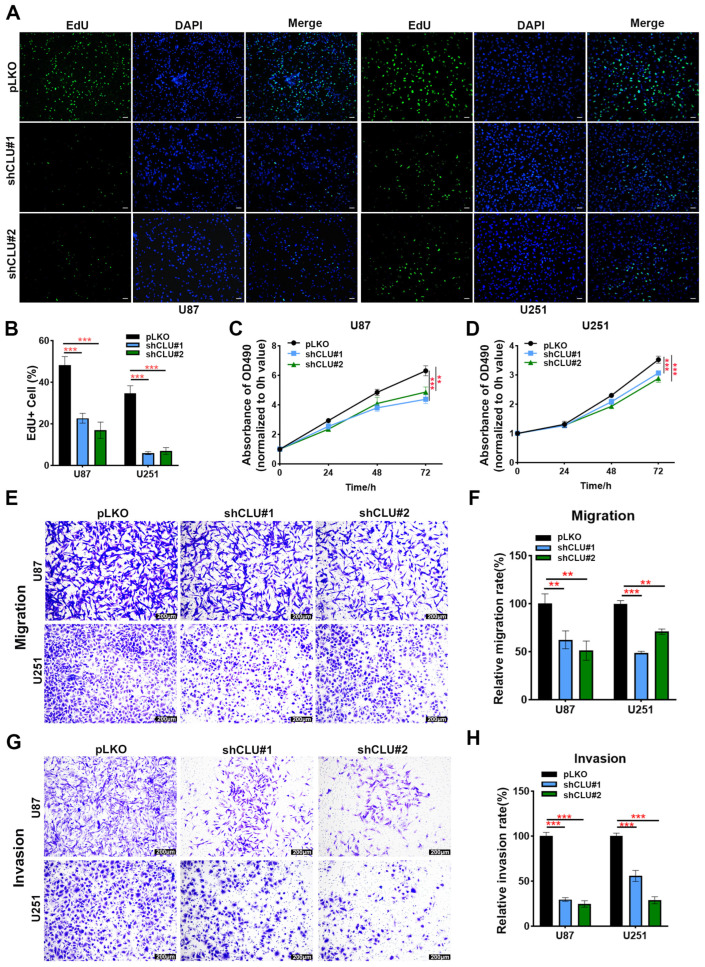
CLU knockdown impairs the proliferative and metastatic ability of GBM cells. (**A**,**B**) The effects of CLU knockdown on cell proliferation in U87 and U251 cells were evaluated by EdU assays. Data are presented as mean ± SD from three independent biological replicates. Statistical significance was determined by two-tailed Student’s *t*-test. ***, *p* < 0.001. (**C**,**D**) The effects of CLU knockdown on cell proliferation in U87 and U251 cells were evaluated by MTT assays. All cells were subjected to MTT analysis at the indicated time points. Data are presented as mean ± SD from three independent biological replicates. Statistical significance was determined by two-way ANOVA followed by Tukey’s post hoc test. **, *p* < 0.01; ***, *p* < 0.001. (**E**,**F**) The effect of CLU knockdown on the migration of U251 and U87 cells using the Transwell assay. Data are presented as mean ± SD from three independent biological replicates. Statistical significance was determined by two-tailed Student’s *t*-test. **, *p* < 0.01; ***, *p* < 0.001. Magnification, ×100. Scale bar, 200 μm. (**G**,**H**) The effect of CLU knockdown on the invasion of U251 and U87 cells using the Transwell assay. Data are presented as mean ± SD from three independent biological replicates. Statistical significance was determined by two-tailed Student’s *t*-test. ***, *p* < 0.001. Magnification, ×100. Scale bar, 200 μm.

To characterize the functional role of CLU in GBM, we transfected U251, U87, and LN229 cells with CLU-targeting shRNAs to knock down endogenous CLU expression and validated the knockdown efficiency via Western blotting (Figure S4A,B). Functional assays showed that CLU knockdown significantly impaired the proliferative capacity of GBM cells, as evidenced by reduced cell viability in MTT assays and decreased EdU-positive cell ratios (Figure 4A–D and Figure S4C–E). Similarly, Transwell migration and invasion assays showed that CLU knockdown significantly attenuated the migratory and invasive capabilities of GBM cells compared with control cells (Figure 4E–H and Figure S4F,G). These results indicate that CLU exerts a pro-malignant function in GBM cells, consistent with the phenotypic effects of COPS7B.

### 2.5. The Malignancy-Driving Function of COPS7B Is Dependent on CLU-Mediated Downstream Signaling

**Figure 5:**
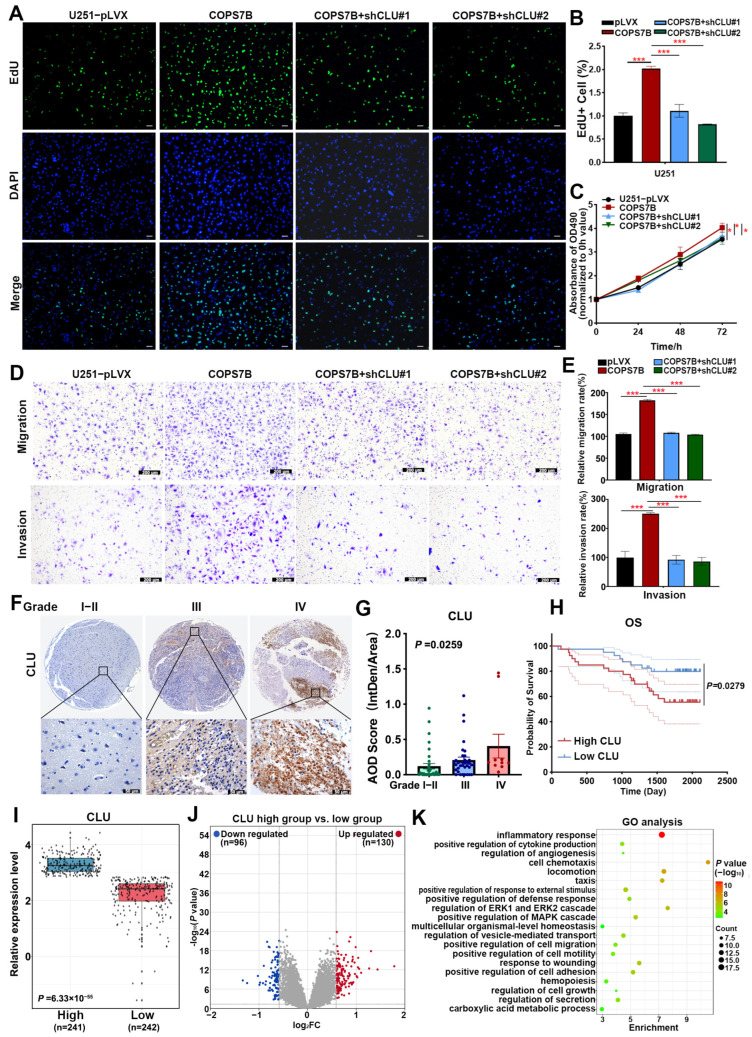
CLU is a key downstream target gene for COPS7B in promoting the malignant phenotype of GBM. (**A**,**B**) The effect of COPS7B overexpression and CLU knockdown on cell proliferation in U251 cells by EdU assays. Data are presented as mean ± SD from three independent biological replicates. Statistical significance was determined by two-tailed Student’s *t*-test. ***, *p* < 0.001. (**C**) Rescued effects of CLU on cell proliferation upon COPS7B overexpression of U251 cells were evaluated by MTT assays. All cells were subjected to MTT analysis at the indicated time points. Data are presented as mean ± SD from 3 independent biological replicates. Statistical significance was determined by two-way ANOVA followed by Tukey’s post hoc test. *, *p* < 0.05. (**D**,**E**) Rescued effects of CLU on migration and invasion of U251 cells upon COPS7B overexpression were evaluated. Data are presented as mean ± SD from three independent biological replicates. Statistical significance was determined by two-tailed Student’s *t*-test. ***, *p* < 0.001. Magnification, ×100. Scale bar, 200 μm. (**F**) Representative IHC staining images of CLU in glioma microarray. Magnification, ×400. Scale bar, 50 μm. (**G**) Quantitative analysis of IHC microarray staining intensity (grade I–II, *n* = 35; III, *n* = 35; IV, *n* = 10). Data are presented as mean ± SD. Statistical significance was determined by one-way ANOVA followed by Tukey’s post hoc test. (**H**) Kaplan–Meier OS analysis showing the association between CLU protein expression and OS of glioma patients in the IHC microarray (Blue lines: low expression group, *n* = 40; red lines: high expression group, *n* = 40). Statistical significance was determined by the log-rank test. (**I**) The RNA expression level of CLU in GBM, CLU high group (*n* = 241) and low group (*n* = 242). (**J**) The volcano plot displays the differential expression alterations of genes in the high-CLU expression group (*n* = 241) compared with the low-CLU expression group (*n* = 242). A total of 130 genes were significantly upregulated, and 96 genes were significantly downregulated. Red indicates upregulated genes, green indicates downregulated genes, and grey indicates genes with no significant differential expression. Statistical significance was determined by two-tailed Student’s *t*-test with Benjamini–Hochberg correction for multiple comparisons. (**K**) GO analysis of up-regulated genes in CLU high group compared with low group.

To verify whether CLU is functionally required for COPS7B-driven malignant phenotypes in GBM, we performed functional rescue assays by knocking down CLU in COPS7B-overexpressing GBM cells. Western blotting confirmed stable overexpression of COPS7B, and the concomitant upregulation of CLU protein was effectively reversed by CLU shRNA transduction (Figure S5A–C). Functional assays showed that CLU silencing significantly abrogated the enhanced proliferation induced by COPS7B overexpression, as consistently demonstrated by MTT and EdU incorporation assays (Figure 5A–C and Figure S5D–F). In parallel, the increased migratory and invasive capacities conferred by COPS7B overexpression were also markedly attenuated by CLU knockdown, with these phenotypes restored to levels comparable to those control cells (Figure 5D,E and Figure S5G,H). Collectively, these findings indicate that CLU is an essential downstream mediator for the oncogenic effects of COPS7B in GBM cells.

To further substantiate the clinical relevance of CLU in glioma progression, we performed IHC on glioma tissue microarrays. In accordance with the expression pattern of COPS7B, CLU protein expression was positively associated with WHO pathological grade, with progressively higher staining intensity observed in higher-grade gliomas (Figure 5F,G). Furthermore, survival analysis based on IHC scoring revealed that GBM patients with high CLU protein abundance had significantly poorer OS than those with low CLU expression (Figure 5H). These clinical data corroborate the pro-malignant role of CLU in glioma and support the clinical significance of the COPS7B-CLU regulatory axis.

To explore the molecular mechanism underlying CLU-mediated GBM progression, we analyzed the TCGA-GBM Agilent microarray dataset downloaded from the UCSC Xena database. Samples were stratified into high- and low-CLU expression groups according to the median expression level of CLU (Figure 5I). Differential expression analysis identified 130 significantly upregulated genes and 96 significantly downregulated genes in the high-CLU group relative to the low-CLU group (Figure 5J; Supplementary Table S4). Functional enrichment analysis showed that the upregulated genes were significantly enriched in the ERK cascade, MAPK signaling cascade, and biological processes associated with cell growth, invasion, and migration (Figure 5K). These findings suggest that the CLU exerts its progression-promoting effects in GBM, at least in part, through the activation of tumor-associated mitogenic signaling pathways.

## 3. Discussion

GBM remains among the most lethal primary malignant tumors of the adult central nervous system, with stagnant clinical outcomes over the past decades despite therapeutic advances, largely due to incomplete elucidation of its core malignancy-driving factors and non-genomic regulatory mechanisms [11,12,13]. In recent years, dysregulated mRNA translation has emerged as a hallmark and critical therapeutic vulnerability of GBM, as it drives the rapid synthesis of oncogenic proteins to fuel malignant progression even in the context of stable transcript levels [14,15,16,17]. In this study, we systematically identified COPS7B as a novel regulator of GBM malignant progression and demonstrated that it promotes GBM cell proliferation, migration, and invasion by specifically enhancing the mRNA translation efficiency of its key downstream effector, CLU.

The canonical function of COPS7B has long been restricted to its role as a structural core subunit of the CSN complex, where it maintains CSN holoenzyme integrity to support the deneddylation of CRLs and subsequent regulation of ubiquitin-mediated protein degradation [18]. For decades, studies on COPS7B in malignancies have almost exclusively focused on this CSN-dependent proteostatic function, with little attention paid to its potential non-canonical activities independent of the CSN complex [19]. Notably, accumulating evidence indicates that translational reprogramming is a pivotal driver of GBM pathogenesis, with most prior work centering on core translation machinery components such as the eIF4F initiation complex and aberrant ribosomal proteins [20,21,22,23]. In contrast to these canonical translation regulators, COPS7B represents a functionally distinct regulatory node originating from the COP9 signalosome. Rather than acting as a general component of the translation apparatus, COPS7B exerts its regulatory effect through direct binding to specific target mRNAs (as demonstrated by our RIP-qPCR data) and modulation of ribosome biogenesis, thereby selectively enhancing the translation of oncogenic transcripts such as CLU. This CSN-independent translational activity distinguishes COPS7B from most other CSN subunits, whose tumor-related functions have been almost exclusively attributed to their canonical deneddylation activity in the ubiquitin–proteasome pathway [7,24,25]. Building on our previous report of COPS7B’s translational regulatory function in other malignancies [8], the present study extends this finding to GBM for the first time, and provides robust evidence that COPS7B drives GBM aggressiveness via this non-canonical axis. Specifically, we show that COPS7B upregulates CLU protein abundance without altering its mRNA transcription or protein stability, and polysome profiling confirms that COPS7B overexpression enhances the translation efficiency of CLU mRNA. These data demonstrate that the malignancy-driving function of COPS7B in GBM is independent of the CSN-mediated proteasome degradation pathway and is attributed to its translational regulatory activity.

CLU is a multifunctional secreted chaperone protein implicated in a wide range of physiological and pathological processes, including neurodegeneration and malignant progression [26,27,28]. Previous studies in glioma have consistently documented a progression-promoting role for CLU, and have linked its oncogenic effects to activation of downstream signaling cascades, including PI3K/Akt and MAPK/ERK pathways, which modulate cell survival, invasion and mesenchymal transition [29,30,31,32,33,34]. In line with these reports, our transcriptomic analysis also identified significant enrichment of ERK and MAPK signaling cascades among genes upregulated by CLU, supporting a conserved pro-malignant function of CLU in GBM. With regard to the regulation of CLU in tumors, prior work has primarily focused on transcriptional activation induced by extracellular stimuli such as TGF-β, oxidative stress and hypoxic conditions [28,35,36]. These transcriptional regulatory mechanisms account for the elevated CLU mRNA levels observed in GBM tissues relative to normal brain tissues, which is consistent with the expression pattern observed in our cohort. In contrast to these well-characterized transcriptional regulatory pathways, our study reveals an additional post-transcriptional regulatory mode for CLU in GBM: COPS7B upregulates CLU protein abundance by enhancing the translation efficiency of CLU mRNA, without affecting its transcription or protein stability. This translational regulatory layer represents a previously unreported mechanism that further augments CLU protein expression in GBM. It complements the known transcriptional upregulation and expands the current understanding of the CLU regulatory network in brain malignancies. From a clinical perspective, these findings indicate that CLU upregulation in GBM is driven by both transcriptional and translational mechanisms. Targeting COPS7B-mediated translational control may provide an alternative strategy to reduce CLU protein expression and suppress its pro-tumor effects, which offers a mechanistic reference for future translational research in GBM.

From a translational perspective, the COPS7B-CLU axis identified in this study provides candidate molecular entry points for further intervention research. As a post-transcriptional regulatory node that controls the expression of multiple oncogenic effector proteins, COPS7B may serve as a potential target for suppressing the malignant progression of GBM. However, we acknowledge that the development of targeted therapeutic agents is a separate and extensive research field, and further in vivo validation, pharmacological development and clinical investigation are required before any clinical application can be realized.

### Study Limitations

This study has several limitations that warrant further investigation. First, the precise molecular mechanism by which COPS7B exerts its CSN-independent translational regulation of CLU mRNA—including whether it directly binds to CLU mRNA via specific cis-elements or interacts with core translation initiation/elongation factors to modulate ribosome recruitment—remains to be fully elucidated. Second, the in vivo function of the COPS7B-CLU axis needs to be validated in immunocompetent orthotopic GBM models, which will better recapitulate the tumor microenvironment of human GBM. Finally, exploring the upstream regulators of COPS7B expression in GBM, and the potential crosstalk between its non-canonical translational function and canonical CSN-dependent proteostatic activity, will provide a more comprehensive understanding of this regulatory node in GBM progression.

## 4. Methods

### 4.1. Data Sources

Public transcriptomic and proteomic datasets were utilized to analyze the expression pattern and clinical relevance of COPS7B in glioma. The mRNA expression profiles of COPS7B across glioma and normal brain tissues were retrieved from the Gene Expression Profiling Interactive Analysis (GEPIA) web server (http://gepia.cancer-pku.cn/detail.php, accessed on 10 November 2024). The RNA sequencing data and matched clinical prognostic information of primary and recurrent glioblastoma samples were downloaded from the Chinese Glioma Genome Atlas (CGGA) database (https://www.cgga.org.cn, accessed on 10 November 2024), which contains large-scale multi-omic data from Chinese glioma cohorts with complete histopathological annotations. Global protein expression data of GBM tissues were obtained from the Clinical Proteomic Tumor Analysis Consortium (CPTAC) via the Proteomic Data Commons portal (https://pdc.cancer.gov/pdc/browse, accessed on 18 March 2025). For CLU regulatory network analysis, Agilent G4502A microarray gene expression data of GBM were downloaded from the UCSC Xena Database (https://xena.ucsc.edu, accessed on 16 April 2026), and subsequent differential expression and functional enrichment analyses were performed based on this dataset.

### 4.2. IHC and Quantitative Scoring

IHC staining was performed to assess the protein expression of COPS7B and CLU in clinical glioma tissues. A human glioma tissue microarray (Cat# BraSur2201) was purchased from Hunan Aifang Biological Co., Ltd. (Changsha, China). The study protocol was reviewed and approved by the Ethics Committee on Biological Science and Technology of Hunan Aifang Biological Co., Ltd. (Approval No. HN20250401). Paraffin-embedded sections were sequentially deparaffinized, rehydrated through graded ethanol, and subjected to heat-induced antigen retrieval in lactate buffer (pH 6.0). Endogenous peroxidase activity and non-specific antigen binding were blocked sequentially, followed by overnight incubation with primary antibodies at 4 °C. Sections were then incubated with matching horseradish peroxidase-conjugated secondary antibodies at 37 °C for 30 min. Protein expression was visualized using 3,3′-diaminobenzidine (DAB) as the chromogenic substrate, and hematoxylin was used for nuclear counterstaining. Quantitative analysis of staining intensity was performed using ImageJ software (version 1.54P, RRID: SCR_003070). The area optical density (AOD) was calculated as the ratio of integrated optical density (IOD) to the total area of the targeted tissue region and was used to compare COPS7B and CLU protein levels across different WHO pathological grades. For prognostic analysis, Kaplan–Meier survival analysis with log-rank test was conducted by correlating AOD values with matched overall survival data from the tissue microarray cohort. All primary and secondary antibodies used in this assay are listed in Supplementary Table S1.

### 4.3. Cell Culture

Four cell lines used in this study: human embryonic kidney 293TN (RRID: CVCL_UL49) cells and three human GBM cell lines U251 (RRID: CVCL_0021), U87 (RRID: CVCL_GP63), and LN229 (CVCL_0393) obtained from Procell (Wuhan, China). All cell lines were cultured in Dulbecco’s modified Eagle’s medium (DMEM) supplemented with 10% fetal bovine serum and 1% penicillin–streptomycin and maintained in a humidified incubator at 37 °C with 5% CO_2_. All cell lines were authenticated via short tandem repeat (STR) profiling and confirmed to be mycoplasma-negative before use. Cells were used for experiments within two months after resuscitation to ensure consistent cellular phenotypes.

### 4.4. Construction of Stable Transfection Cell Lines

Lentiviral vectors were constructed to establish stable cell lines with target gene overexpression or knockdown. For overexpression constructs, the full-length coding sequences (CDS) of COPS7B were inserted into the pLVX plasmid. For knockdown constructs, chemically synthesized short hairpin RNA (shRNA) sequences targeting COPS7B and CLU were cloned into the pLKO.1 plasmid. All recombinant plasmids were verified by Sanger sequencing. To produce lentiviral particles, the constructed expression plasmids were co-transfected into 293TN cells together with the packaging plasmids pMD2.G and PsPAX2. Viral supernatants were collected 48 h and 72 h after transfection, filtered, and used to infect three glioma cell lines following a previously described protocol [37]. Stably transfected cell populations were selected and maintained with puromycin at an appropriate concentration. The sequences of PCR primers and shRNA used in this study are listed in Supplementary Table S1.

### 4.5. Cell Proliferation Assays

Cell proliferative capacity was evaluated using 3-(4,5-dimethylthiazol-2-yl)-2,5-diphenyltetrazolium bromide (MTT) and 5-ethynyl-2′-deoxyuridine (EdU) incorporation assays. For the MTT assay, cells in the logarithmic growth phase were seeded into 96-well plates at a density of 4 × 10^3^ cells per well and cultured for 24, 48, and 72 h. At each time point, 20 μL of 5 mg/mL MTT solution was added to each well and incubated for 4 h at 37 °C. After removing the supernatant, 100 μL of dimethyl sulfoxide (DMSO) was added to each well to dissolve the formazan crystals, followed by incubation for 10 min at 37 °C. The absorbance at 492 nm was measured using a microplate reader, and cell viability was calculated as the ratio of absorbance at each time point to that at the 0 h baseline.

The EdU assay was performed using the EdU kit (Beyotime Biotechnology, Dalian, China), according to the manufacturer’s instructions. Briefly, cells were seeded into 24-well plates with coverslips and cultured until adhered, then incubated with EdU working solution for 2 h. After fixation with 4% paraformaldehyde for 15 min and washing with a 3% BSA solution, cells were permeabilized and incubated with the click reaction mixture in the dark for 10 min. Cell nuclei were counterstained with Hoechst 33342. Images were captured under a fluorescence microscope (Nikon, Tokyo, Japan), and the proportion of EdU-positive cells was quantified to reflect DNA synthesis activity.

### 4.6. Transwell Cell Migration and Invasion Assay

The migratory and invasive capacities of GBM cells were assessed using Transwell chambers with 8 μm pores (3422, Corning, NY, USA). For the migration assay, cells were resuspended in serum-free DMEM and seeded into the upper chamber at a density of 5 × 10^4^ cells per 300 μL, while 700 μL of complete DMEM containing 10% FBS was added to the lower chamber as a chemoattractant. After incubation for 6 h (U251 and LN229 cells) or 24 h (U87 cells), cells remaining on the upper surface of the membrane were gently removed with cotton swabs, and cells that migrated to the lower surface were fixed and stained with 0.5% crystal violet solution at room temperature. For the invasion assay, the upper surface of the Transwell membrane pre-coated with Matrigel to mimic the extracellular matrix, and 1 × 10^5^ cells were seeded into each upper chamber; all other procedures were identical to those of the migration assay. After staining, images of five randomly selected fields were captured under a microscope, and the number of migrated or invaded cells was quantified using ImageJ software.

### 4.7. Western Blot

Total protein was extracted from cultured cells using RIPA lysis buffer (Beyotime Biotechnology, China) supplemented with a protease inhibitor cocktail (Meilun Biotechnology, Dalian, China). Cells were lysed on ice for 30 min, followed by centrifugation at 12,000 *g* for 15 min at 4 °C to collect the supernatant. Protein concentration was determined using a BCA protein assay kit (Beyotime Biotechnology, China). Equal amounts of protein samples were separated by SDS-PAGE gels electrophoresis and transferred onto polyvinylidene difluoride (PVDF) membranes (Sigma-Aldrich, Merck KGaA, Taufkirchen, Germany). The membranes were blocked with 5% non-fat milk for 1 h at room temperature, then incubated with the primary antibodies overnight at 4 °C. After washing with TBST, the membranes were incubated with the appropriate secondary antibody for 1 h at room temperature. Protein bands were visualized using an enhanced chemiluminescence detection system, and band intensities were quantified using ImageJ software. The antibodies used in this study are provided in Supplementary Table S1.

### 4.8. Reverse Transcription and Real-Time Fluorescent Quantitative PCR (RT-qPCR)

Total RNA was extracted from cells using Trizol (Invitrogen, Carlsbad, CA, USA), and the reaction system was prepared according to the PrimeScript RT Master Mix kit (Takara, Tokyo, Japan) for reverse transcription. The reaction was set at 37 °C for 15 min and terminated at 85 °C. The obtained cDNA was subjected to real-time quantitative PCR analysis using LC96 (Roche, Basel, Switzerland). The normalization of all target gene expressions was performed using GAPDH as an internal reference. The relative mRNA expression levels were quantified using the 2^−ΔΔCt^ method. The primer sequences are shown in Supplementary Table S1.

### 4.9. Protein Stability and Protein Synthesis Assays

To evaluate the effect of COPS7B on CLU protein stability, cells in the COPS7B overexpression and control groups were seeded into 6-well plates and grown to 40% confluence, then treated with the protein synthesis inhibitor cycloheximide (CHX, 100 μg/mL, Cat#S7418, Selleckchem, Houston, TX, USA) for 0, 3, 6, and 9 h. Cells were harvested at each time point for protein extraction and Western blot analysis. The degradation rate of CLU protein was calculated by normalizing its band intensity at each time point to that at the 0 h time point.

To assess de novo protein synthesis, cells were seeded as described above and treated with the proteasome inhibitor MG132 (10 μM, Cat#HY-13259, MedChemExpress LLC, South Brunswick, NJ, USA) for 0, 3, 6, and 9 h. Cells were collected at each time point for Western blot detection. The accumulation rate of CLU protein under proteasome inhibition was used to reflect its de novo synthesis efficiency. All band intensities were quantified using ImageJ software.

### 4.10. RIP Assay

The RIP assay was performed to detect the binding interaction between COPS7B protein and endogenous CLU mRNA, following a previously established protocol [38]. Briefly, a total of 1 × 10^7^ U251 cells were harvested and resuspended in 400 μL of ice-cold RIP lysis buffer containing 25 mmol/L Tris-HCl (pH 7.5), 150 mmol/L KCl, 2 mmol/L EDTA, 0.5% NP-40, 1 mmol/L NaCl, 1 mmol/L DTT, 100 U/mL RNasin ribonuclease inhibitor, and EDTA-free protease inhibitor cocktail. Cells were lysed on ice for 30 min, followed by centrifugation at 16,000× *g* for 10 min at 4 °C to obtain clarified cell lysate supernatant. Protein A/G magnetic beads (Invitrogen, CA, USA) were pre-equilibrated with lysis buffer and incubated with the corresponding primary antibody (Supplementary Table S1) or normal rabbit IgG isotype control (Proteintech Cat# 30000-0-AP, RRID: AB_2819035) with end-over-end rotation at 4 °C for 6 h to generate antibody–bead complexes. The prepared complexes were then incubated with the cell lysate supernatant overnight at 4 °C with constant rotation for immunoprecipitation. After incubation, beads were collected by brief centrifugation at 1000 rpm for 10 s at 4 °C, and the supernatant was discarded. The beads were washed twice with ice-cold low-salt wash buffer (5 min per wash), followed by two additional washes with ice-cold high-salt wash buffer (5 min per wash). The low-salt wash buffer consisted of 50 mmol/L Tris-HCl (pH 7.4), 150 mmol/L NaCl, 1 mmol/L MgCl_2_, 0.05% NP-40, 2 mmol/L EDTA, 1 mmol/L DTT, and 100 U/mL RNasin ribonuclease inhibitor. Finally, the beads were resuspended in Proteinase K digestion buffer and incubated at 55 °C for 10 min to digest bead-bound proteins and release immunoprecipitated RNA. Total RNA was extracted from the eluted products using the standard TRIzol reagent method, followed by ethanol precipitation and reverse transcription into cDNA. The relative enrichment of CLU mRNA in COPS7B immunoprecipitates was quantified via RT-qPCR, with all primer sequences provided in Supplementary Table S1.

### 4.11. Polysome Profiling

Polysome profiling was performed to assess global mRNA translation efficiency. Briefly, cells were pre-treated with CHX (100 μg/mL) for 10 min at 37 °C to arrest translating ribosomes on mRNA, then harvested by a trypsinization and washed with ice-cold PBS containing CHX. Cells were lysed in polysome lysis buffer (20 mmol/L Tris-HCl, pH 7.4, 5 mmol/L MgCl_2_, 150 mmol/L NaCl, 1% Triton X-100, RNase inhibitor, protein inhibitor, 100 μg/mL CHX, 1 mmol/L DTT) on ice for 15 min, followed by centrifugation at 15,000 rpm for 15 min at 4 °C. The supernatant was loaded onto a pre-prepared 10–50% linear sucrose gradient and centrifuged at 38,000 rpm for 3 h. Gradient fractions were collected using a Gradient Station (BioComp, Fredericton, NB, Canada), and absorbance at 254 nm was continuously monitored with ECONO UV detector (BioComp, Canada). The ratio of polysome area to monosome area was calculated to reflect global translation efficiency.

### 4.12. Liquid Chromatograph Mass Spectrometer (LC-MS)

Comparative quantitative proteomic profiling was performed to identify downstream effectors of COPS7B in GBM cells. The LC-MS detection and corresponding bioinformatic analyses in this study were completed by PTM Biolabs (Hangzhou, China). Cells were lysed with lysis buffer containing 8 M urea and 1% protease inhibitor, followed by the addition of 5 mM DTT and 11 mM iodoacetamide. The proteins were then digested with trypsin, quantified, and combined with GST and MBP standard proteins in equal amounts. Peptides were dissolved in 0.1% formic acid and loaded into an EASY-nLC 1200 ultra-high-performance liquid chromatography system. Peptides were analyzed on an Orbitrap Exploris 480 mass spectrometer, and data were collected using the data-independent acquisition (DIA) program. Gene Ontology (GO) functional enrichment analysis of differentially expressed proteins was conducted using the Metascape platform (https://metascape.org, accessed on 18 March 2025). The proteomic data are summarized in Supplementary Table S2.

### 4.13. CLU Regulatory Network Analysis

To explore the downstream signaling pathways mediated by CLU in GBM, we downloaded Agilent G4502A microarray expression data of GBM from the UCSC Xena Database and performed data preprocessing using R version 4.3.0. Samples were divided into high- and low-CLU expression groups according to the median expression level of CLU. Differential expression analysis between the two groups was conducted using the limma package: a linear model was fitted via the lmFit function, between-group expression differences were estimated using the contrasts.fit function, and empirical Bayes moderation of the standard errors was performed with the eBayes function to improve statistical power. The screening criteria were set as an adjusted *p*-value < 0.05 and an absolute log2 fold change (|log_2_FC|) > 0.585, under which significantly differentially expressed genes between the two groups were identified. The full list of differentially expressed genes is provided in Supplementary Table S4.

### 4.14. Statistical Analysis

All quantitative experimental data are presented as mean ± standard deviation (SD), and all statistical analyses were performed using GraphPad Prism 9.0 software. The Shapiro–Wilk test was first applied to assess the normality of data distribution for all datasets prior to hypothesis testing. For datasets conforming to a normal distribution, parametric statistical methods were adopted: statistical significance between two independent groups was determined using a two-tailed Student’s *t*-test; for comparisons across three or more experimental groups, one-way analysis of variance (ANOVA) was performed, followed by Tukey’s post hoc test to correct for multiple comparisons. For datasets that did not follow a normal distribution, nonparametric tests were used: the Mann–Whitney U test was applied for two-group comparisons, and the Kruskal–Wallis test was applied for multi-group comparisons. For survival analysis, Kaplan–Meier curves were constructed, and between-group differences in overall survival were evaluated using the log-rank test. *p* < 0.05 was considered statistically significant.

## 5. Conclusions

In summary, our study identifies COPS7B as a novel regulator of GBM malignant progression and demonstrates that its progression-promoting effects are exerted through a CSN-independent translational regulatory function, rather than its canonical proteasome-related activity. We further delineate the COPS7B-CLU translational regulatory axis as a key pathway driving GBM malignant progression. These findings expand the understanding of CSN subunits’ function in GBM and provide candidate prognostic biomarkers and mechanistic targets for future translational research in GBM.

## Data Availability

Publicly available datasets analyzed in this study were obtained from GEPIA, CGGA, CPTAC, and the UCSC Xena database. All other data supporting the findings of this study are available from the corresponding author upon reasonable request.

## Supplementary Materials

The following supporting information can be downloaded at: https://www.mdpi.com/article/10.3390/ijms27156976/s1.
