# Supplementary material for: COPS7B Drives Malignant Progression of Glioblastoma Through Translational Upregulation of the Downstream Functional Effector CLU
Source: Int J Mol Sci. 2026 Aug 3;27(15):6976. doi: 10.3390/ijms27156976 (PMC13467472; doi:10.3390/ijms27156976)
Supplement: Supplementary file 1 [file ijms-27-06976-s001.zip › Supplementary figures.pdf]

Supplementary figures

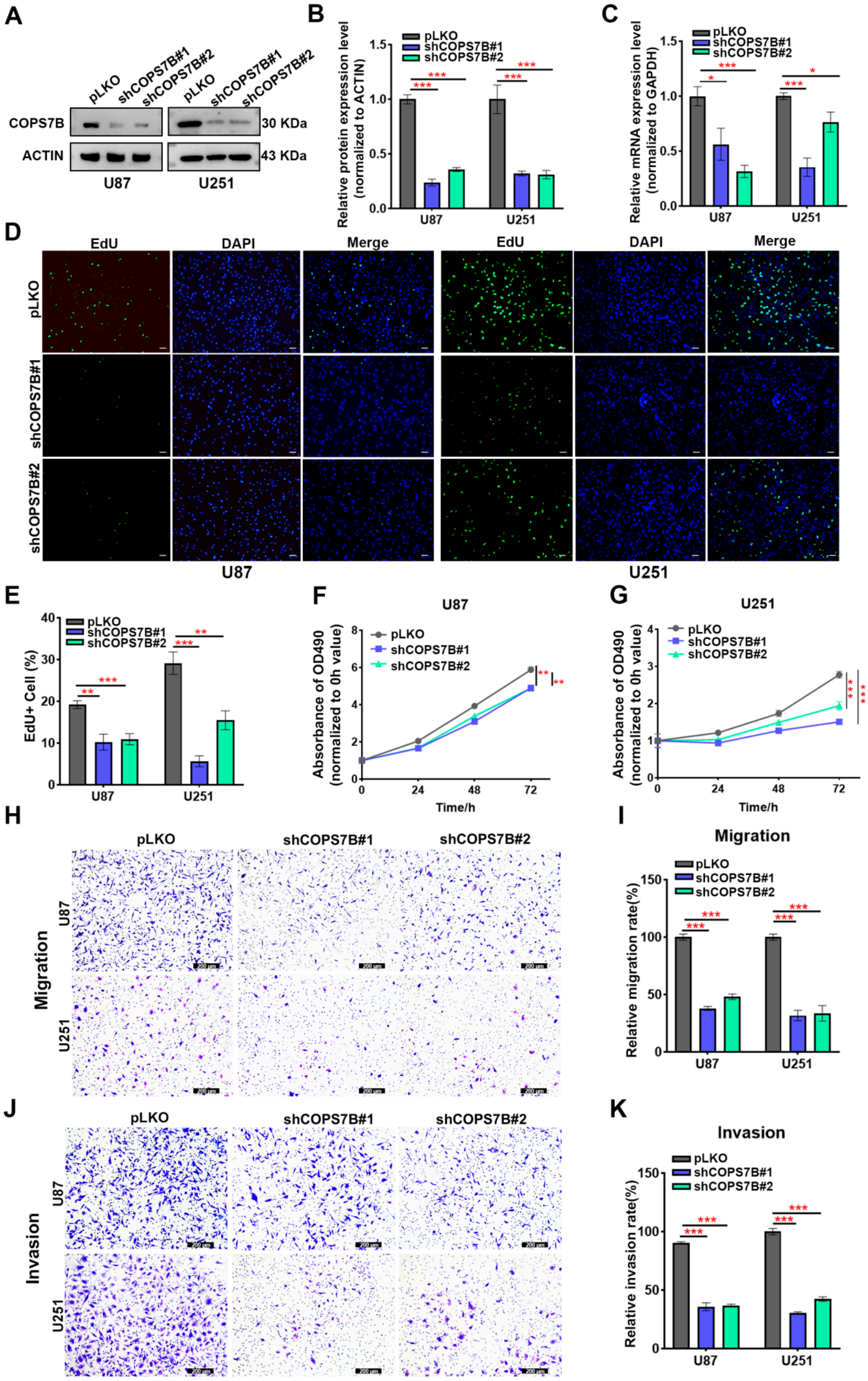

Figure S1. COPS7B knockdown impairs the proliferative and metastatic ability of GBM cells

A. Western blotting analysis of COPS7B protein levels in COPS7B-knockdown and control U251 and U87 cells.

B. Western Blot grayscale value quantitatively detected the expression of COPS7B in U251 and U87 cell lines. Quantifications of Western blotting include data from at least three biological replicates. Data are presented as mean ± SD. Statistical analysis was performed using two-tailed Student's *t*-test. *** , $P < 0.001$.

C. RT-qPCR detected the expression level of COPS7B mRNA in U251 and U87 cell lines. Data are presented as mean ± SD from n = 3 independent biological replicates. Statistical analysis was performed using two-tailed Student's *t*-test. *, $P < 0.05$; *** , $P < 0.001$.

D-E. The effects of COPS7B knockdown on cell proliferation in U87 and U251 cells were evaluated by EdU assays. The EdU-positive rate was calculated as (EdU-positive cells / total cells)× 100%. For each biological replicate, 8 randomly selected fields per group were counted, and the mean positive rate of these fields was used as the replicate-level datum. Data are presented as mean ± SD from n = 3 independent biological replicates. Statistical analysis was performed using two-tailed Student's *t*-test. **, $P < 0.01$; *** , $P < 0.001$.

F-G. The effects of COPS7B knockdown on cell proliferation in U87 and U251 cells were evaluated by MTT assays. All cells were subjected to MTT analysis at the indicated time points. Data are presented as mean ± SD from n = 3 independent biological replicates.Statistical analysis was performed using one-way ANOVA followed by Tukey's post hoc test for each time point. ** , $P < 0.01$; *** , $P < 0.001$.

H-I. The effect of COPS7B knockdown on the migration of U251 and U87 cells using the Transwell assay. Data are presented as mean ± SD from n = 3 independent biological replicates. Statistical analysis was performed using two-tailed Student's *t*-test. Magnification: ×100. Scale bar, 200 μm. ***, $P < 0.001$.

J-K. The effect of COPS7B knockdown on the invasion of U251 and U87 cells using the Transwell assay. Data are presented as mean ± SD from n = 3 independent biological replicates. Statistical analysis was performed using two-tailed Student's *t*-test. Magnification: ×100. Scale bar, 200 μm. *** , $P < 0.001$.

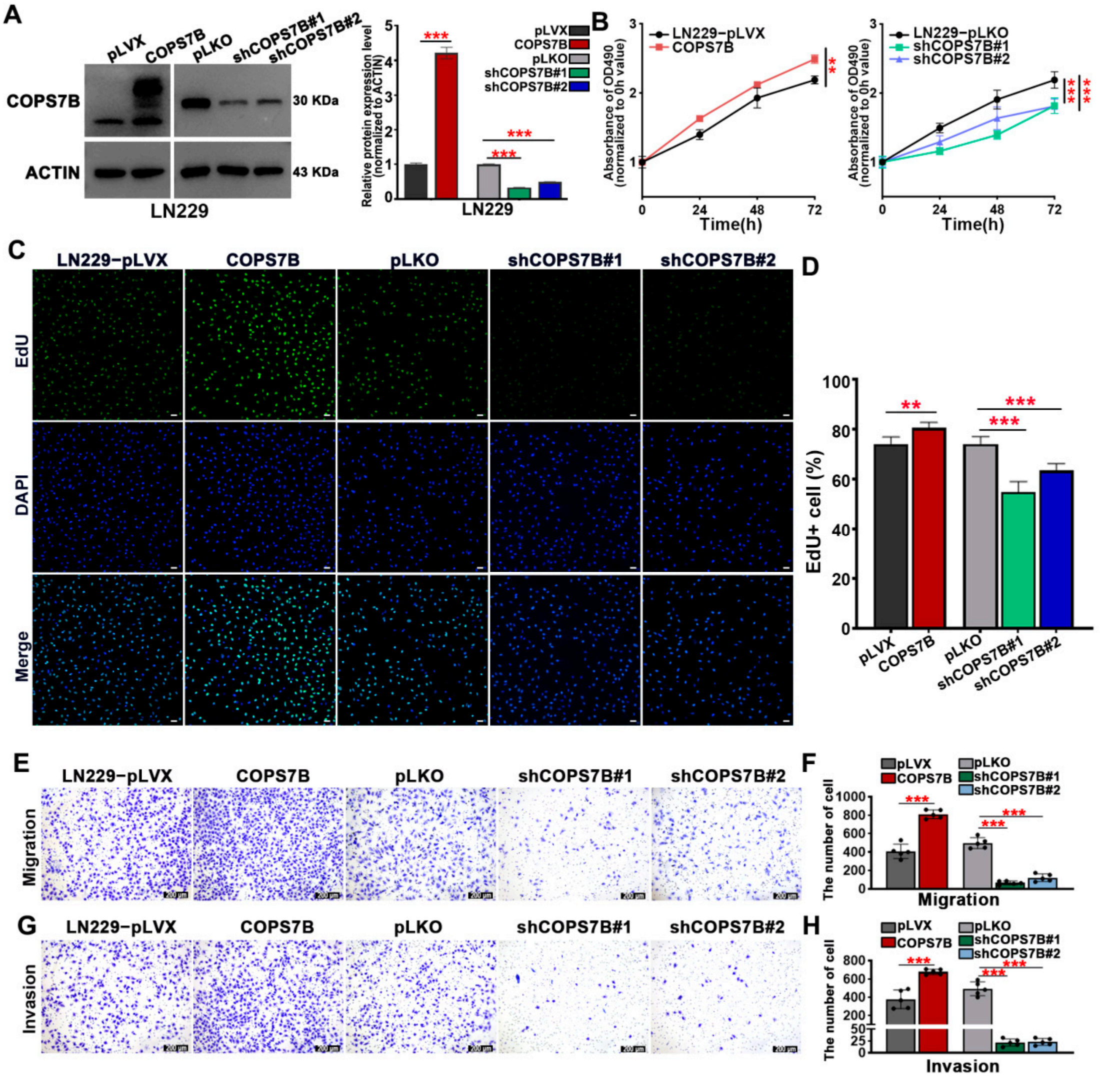

Figure S2. COPS7B influences the proliferative and metastatic ability of LN229 cells

A. Western blotting analysis and densitometric quantification of COPS7B protein levels in LN229 cells with COPS7B overexpression or knockdown. Data are from 3 independent biological replicates and presented as mean ± SD. Statistical analysis was performed using two-tailed Student's *t*-test. *** , $P < 0.001$.

B. The effects of COPS7B overexpression and knockdown on cell proliferation in LN229 cells were evaluated by MTT assays. All cells were subjected to MTT analysis at the indicated time points. Data are presented as mean ± SD from n = 3 independent biological replicates. Statistical analysis was performed using one-way ANOVA followed by Tukey's post hoc test for each time point. ** , $P < 0.01$; *** , $P < 0.001$.

C-D. The effects of COPS7B overexpression and knockdown on cell proliferation in LN229 cells were evaluated by EdU assays. The EdU-positive rate was calculated as (EdU-positive cells / total cells)×100%. For each biological replicate, 8 randomly selected fields per group were counted, and the mean positive rate of these fields was used as the replicate-level datum. Data are presented as mean ± SD from n = 3 independent biological replicates. Statistical analysis was performed using two-tailed Student's *t*-test. **, $P < 0.01$; *** , $P < 0.001$.

E-F. The effect of COPS7B overexpression and knockdown on the migration of LN229 cells using

the Transwell assay. Data are presented as mean ± SD from n = 3 independent biological replicates. Statistical analysis was performed using two-tailed Student's *t*-test. Magnification: ×100. Scale bar, 200 μm. ***, $P < 0.001$.

G-H. The effect of COPS7B overexpression and knockdown on the invasion of LN229 cells using the Transwell assay. Data are presented as mean ± SD from n = 3 independent biological replicates. Statistical analysis was performed using two-tailed Student's *t*-test. Magnification: ×100. Scale bar, 200 μm. *** , $P < 0.001$.

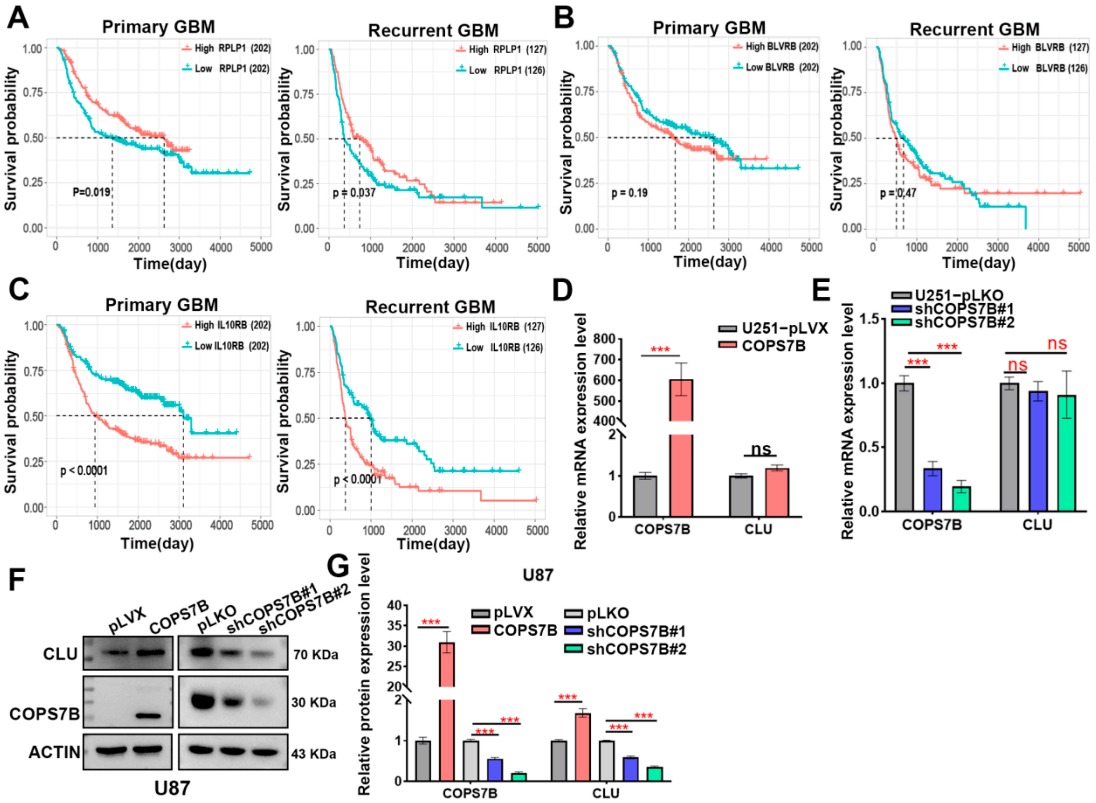

Figure S3. Validation of candidate downstream targets of COPS7B in GBM cells

A-C. Kaplan-Meier survival analysis showing the association between RPLP1, BLVRB and IL10RB expression and OS in patients with primary GBM, respectively. Statistical significance was assessed by the log-rank test.

D-E. RT-qPCR analysis of CLU mRNA levels in U251 cells with COPS7B overexpression or knockdown. Data are presented as mean ± SD from n = 3 independent biological replicates. Statistical analysis was performed using two-tailed Student's *t*-test. ***, $P < 0.001$.

F-G. Western blotting analysis and densitometric quantification of CLU and COPS7B protein levels in U87 cells with COPS7B overexpression or knockdown. Data are presented as mean ± SD from n = 3 independent biological replicates. Statistical analysis was performed using two-tailed Student's *t*-test. ***, $P < 0.001$.

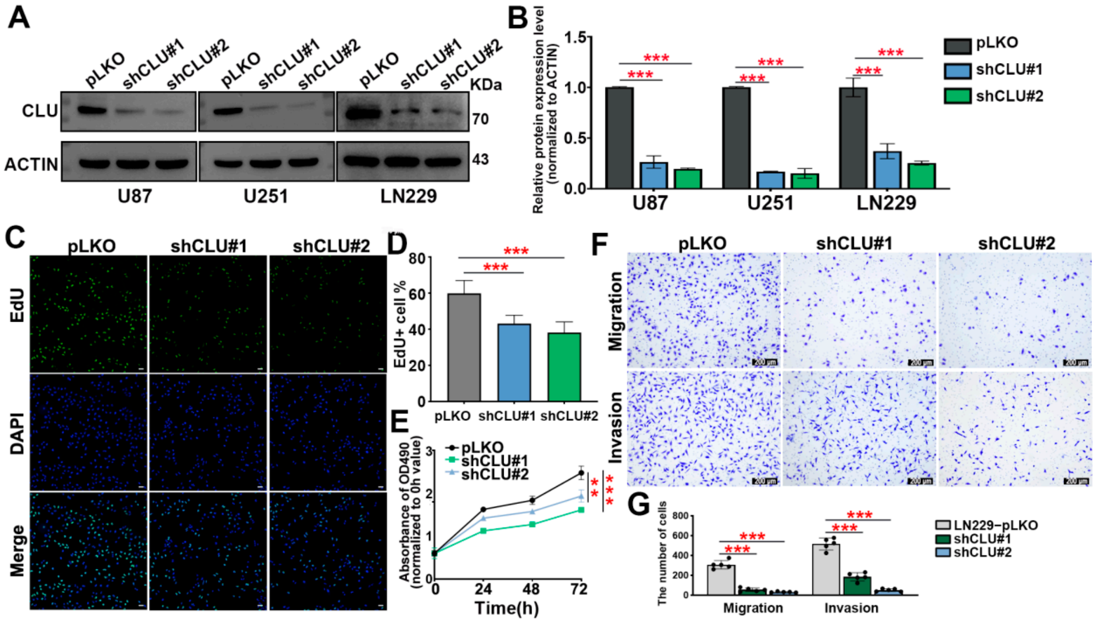

Figure S4. Validation of CLU knockdown efficiency and functional effects in GBM cells

A. Western blotting analysis of CLU protein levels in three GBM cell lines.

B. Western Blot grayscale value quantitatively detected the expression of CLU in three GBM cell lines. Quantifications of Western blotting include data from at least three biological replicates. Data are presented as mean ± SD. Statistical analysis was performed using two-tailed Student's *t*-test. *** , $P < 0.001$.

C-D.The effects of CLU knockdown on cell proliferation in LN229 cells were evaluated by EdU assays. The EdU-positive rate was calculated as (EdU-positive cells / total cells)×100%. For each biological replicate, 8 randomly selected fields per group were counted, and the mean positive rate of these fields was used as the replicate-level datum. Data are presented as mean ± SD from n = 3 independent biological replicates. Statistical analysis was performed using two-tailed Student's *t*-test. *** , $P < 0.001$.

E.The effects of CLU knockdown on cell proliferation in LN229 cells were evaluated by MTT assays. All cells were subjected to MTT analysis at the indicated time points. Data are presented as mean ± SD from n = 3 independent biological replicates. Statistical analysis was performed using one-way ANOVA followed by Tukey's post hoc test for each time point. ** , $P < 0.01$; *** , $P < 0.001$.

F-G. The effect of CLU knockdown on the migration and invasion of LN229 cells using the Transwell assay. Data are presented as mean ± SD from n = 3 independent biological replicates. Statistical analysis was performed using two-tailed Student's *t*-test. Magnification: ×100. Scale bar, 200 μm. **, $P < 0.01$; ***, $P < 0.001$.

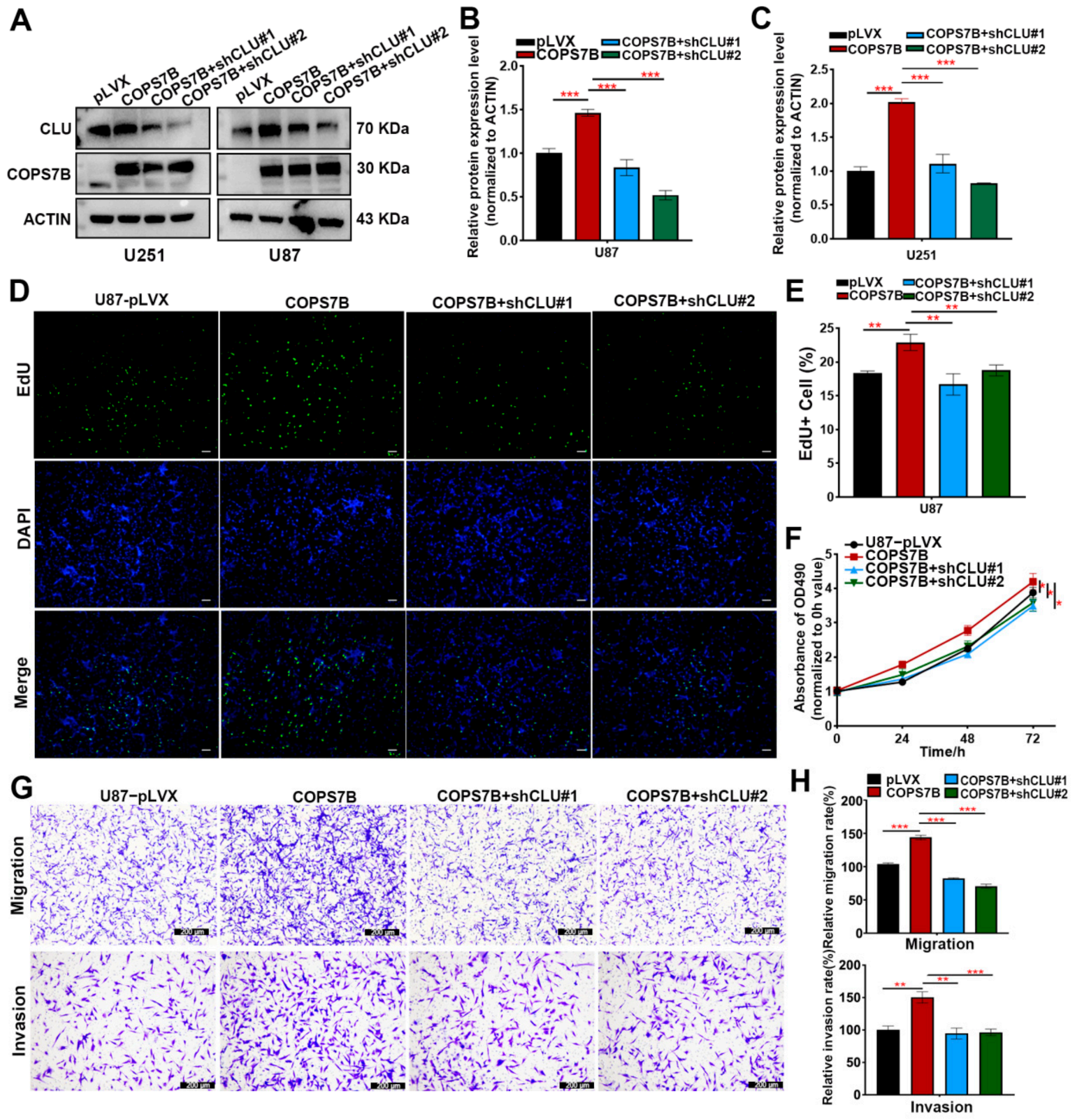

Figure S5. CLU is a key downstream target gene for COPS7B in promoting the malignant phenotype of GBM

A-C. Western blotting analysis and densitometric quantification of CLU and COPS7B protein levels in U251 and U87 cells with COPS7B overexpression alone or combined with CLU knockdown. CLU levels were normalized to ACTIN. Data are from 3 independent biological replicates and presented as mean ± SD. Statistical analysis was performed using two-tailed Student's *t*-test.*** , $P < 0.001$.

D-E. The effect of COPS7B overexpression and CLU knockdown on cell proliferation in U87 cells by EdU assays. The EdU-positive rate was calculated as (EdU-positive cells / total cells)×100%. For each biological replicate, 8 randomly selected fields per group were counted, and the mean positive rate of these fields was used as the replicate-level datum. Data are presented as mean ± SD from n = 3 independent biological replicates. Statistical analysis was performed using two-tailed Student's *t*-test. **, $P < 0.01$.

F. Rescued effects of CLU on cell proliferation upon COPS7B overexpression of U87 cells was evaluated by MTT assays. All cells were subjected to MTT analysis at the indicated time points. Data are presented as mean ± SD from n = 3 independent biological replicates. Statistical analysis

was performed using one-way ANOVA followed by Tukey's post hoc test for each time point. * , $P < 0.05$.

G-H. Rescued effects of CLU on migration and invasion of U87 cells upon COPS7B overexpression were evaluated. Data are presented as mean ± SD from n = 3 independent biological replicates. Statistical analysis was performed using two-tailed Student's *t*-test. Magnification: ×100. Scale bar, 200 μm. **, $P < 0.01$; ***, $P < 0.001$.
